# A Deep Feature Fusion of Improved Suspected Keratoconus Detection with Deep Learning

**DOI:** 10.3390/diagnostics13101689

**Published:** 2023-05-10

**Authors:** Ali H. Al-Timemy, Laith Alzubaidi, Zahraa M. Mosa, Hazem Abdelmotaal, Nebras H. Ghaeb, Alexandru Lavric, Rossen M. Hazarbassanov, Hidenori Takahashi, Yuantong Gu, Siamak Yousefi

**Affiliations:** 1 Biomedical Engineering Department, Al-Khwarizmi College of Engineering, University of Baghdad, Baghdad 10011, Iraq; 2 School of Mechanical, Medical, and Process Engineering, Queensland University of Technology, Brisbane, QLD 4000, Australia; 3 ARC Industrial Transformation Training Centre—Joint Biomechanics, Queensland University of Technology, Brisbane, QLD 4000, Australia; 4 Department of Physics, College of Science, Al-Nahrain University, Baghdad 64021, Iraq; 5 Department of Ophthalmology, Assiut University, Assiut 71526, Egypt; 6 Computers, Electronics and Automation Department, Stefan cel Mare University of Suceava, 720229 Suceava, Romania; 7 Medical School, Universidade Anhembi Morumbi, São Paulo 03101-001, Brazil; 8 Department of Ophthalmology and Visual Sciences, Paulista Medical School, Federal University of São Paulo, São Paulo 04021-001, Brazil; 9 Department of Ophthalmology, Jichi Medical University, Tochigi 329-0431, Japan; 10 Department of Ophthalmology, University of Tennessee Health Science Center, Memphis, TN 38163, USA; 11 Department of Genetics, Genomics, and Informatics, University of Tennessee Health Science Center, Memphis, TN 38163, USA

**Keywords:** convolutional neural networks, keratoconus, feature fusion, transfer learning, deep learning, machine learning

## Abstract

Detection of early clinical keratoconus (KCN) is a challenging task, even for expert clinicians. In this study, we propose a deep learning (DL) model to address this challenge. We first used Xception and InceptionResNetV2 DL architectures to extract features from three different corneal maps collected from 1371 eyes examined in an eye clinic in Egypt. We then fused features using Xception and InceptionResNetV2 to detect subclinical forms of KCN more accurately and robustly. We obtained an area under the receiver operating characteristic curves (AUC) of 0.99 and an accuracy range of 97–100% to distinguish normal eyes from eyes with subclinical and established KCN. We further validated the model based on an independent dataset with 213 eyes examined in Iraq and obtained AUCs of 0.91–0.92 and an accuracy range of 88–92%. The proposed model is a step toward improving the detection of clinical and subclinical forms of KCN.

## 1. Introduction

Keratoconus (KCN) is a non-inflammatory disease that causes thinning and eventual bulging of the cornea affecting one or both eyes [1], and can lead to blindness if not detected and treated at an early stage. KCN detection at an early stage may improve treatment outcomes and prevent development of more advanced stages. Clinical diagnosis of KCN involves the subjective evaluation of the patient’s history, demographic characteristics, and corneal topographic maps [2,3]. While diagnosing established KCN cases can be more straightforward, detecting subclinical forms of KCN remains a challenge.

Machine learning (ML) algorithms [4] have been used as a basis for creating several different automated models, including support vector machines (SVM) [5], decision trees [6], and neural networks [7], which hold promise for the early detection and identification of subclinical forms of KCN, as well as established KCN. These methods are typically combined with hand crafted features [8] or machine generated features from corneal topography. In contrast, deep learning (DL) methods provide a way of both performing feature extraction and classification of images; thus, reducing the need for hand crafted features. DL methods [9,10,11,12] have previously been applied to corneal topography [9,13,14,15,16,17] and optical coherence tomography (OCT) imaging [18] for KCN detection.

One such DL model [15] was developed for KCN detection in patients with a history of refractive surgery from Orbscan corneal maps. A high accuracy of the model was obtained for distinguishing normal from established KCN; however, their model did not include the analysis of suspected KCN. Using a different approach, Al-Timemy et al. [2] developed an ensemble of DL of four corneal maps and Pentacam indices (PI) classifiers for established KCN detection versus normal cases using Pentacam corneal topographic maps. The developed ensemble achieved an accuracy of 95% based on AlexNet. The accuracy was improved to 98.3%, with ensemble-specific combinations of corneal maps’ classifiers and PI. In a third approach, Chen et al. [19] utilized a VGG16 convolutional neural network (CNN) model to detect established KCN versus healthy eyes. Their method was applied to the four topographic maps and achieved an accuracy of 97.85% on the testing set when concatenating all maps. However, no suspect KCN group was included. Abdelmotaal et al. [14] also developed a CNN architecture to discriminate suspect, established KCN, and normal eyes. They obtained an accuracy of 98%, for discriminating normal, suspect KCN, and KCN, based on analyzing four corneal maps.

While the previous studies utilized Pentacam maps, other attempts have been developed on synthetic data. In one such study [20], the authors developed a deep learning model based on CNN synthesized cornel maps and achieved a high accuracy, but the images were synthetic and no suspected KCN was included in their dataset.

Detecting subclinical KCN is challenging for expert clinicians, as there may be no clear manifestation of signs. This problem is even more challenging for less experienced corneal experts. Hence, DL models may play an important role in detecting suspect (subclinical) KCN [21].

In this study, our aim was to improve both the accuracy and robustness of detecting subclinical forms of KCN, based on an ensemble of DL features, which is an emerging approach in which the features are extracted from a pretrained DL network and fed into a machine learning classifier, such as SVM, to make the final classification. This approach has previously been applied to detect two corneal classes (normal and KCN) with four corneal topographic maps and Alexnet with SVM [22], and has also been examined in three-class detection (normal, KCN, and suspect KCN) with seven corneal topographic maps and EfficientNet-b0 architecture and SVM [23].

In order to harvest the power of both DL features and ML classifier, we propose a novel fusion DL method based on Xception [24] and InceptionResNetV2 [25] to achieve a three-class clinical suspect KCN detection. Two datasets of 4752 images were used in this study. A dataset of 4113 images, collected in Egypt, were utilized to develop the fusion-based model, and validated with an independent test set of 639 images acquired in Iraq.

## 2. Materials and Methods

### 2.1. Datasets

Two datasets were used in this study: the first dataset was acquired with the Pentacam instrument in Egypt and the second dataset was collected with the Pentacam instrument in Iraq. Both studies were conducted according to the ethical codes of the declaration of Helsinki.

The first dataset included images from normal, suspect, and established keratoconus, which were also utilized in Abdolmotaal et al. [14]. It included refractive corneal maps, as well as sagittal (Sag), corneal pachymetry (CorPac) and elevation front (ElvF) maps, acquired from the Pentacam instruments (Oculus GmbH, Wetzlar, Germany). This original study was a retrospective review of the Pentacam four-map selectable display images of non-consecutive refractive surgery candidates, patients with unilateral or bilateral keratoconus, and patients with subclinical keratoconus. All study participants provided written consent. Two experienced corneal specialists independently classified the anonymized images as keratoconus, subclinical keratoconus, or normal. The keratoconus class (KCN) included those with a clinical diagnosis of keratoconus (e.g., the presence of a central protrusion of the cornea with Fleicher ring, Vogt striae, or both, as determined by slit-lamp examination) or an irregular cornea (as determined by distorted keratometry mires or distortion of retinoscopic red reflex, or both). The keratoconus class also included the subsequent topographic findings, as summarized by Piñero and colleagues: a focal steepening located in a zone of otrusion, surrounded by concentrically decreasing power zones; focal areas with diopteric (D) values > 47.0 D; inferior–superior (I–S) asymmetry measured as >1.4 D; or angling of the hemimeridians in an asymmetric or broken bowtie pattern, with skewing of the steepest radial axis. The suspect keratoconus class (suspect) included subtle corneal topographic changes within the aforementioned keratoconus abnormalities, with the absence of slit-lamp or visual changes typical of keratoconus (former fruste, or asymptomatic, keratoconus). The traditional class (normal) included refractive surgery candidates and subjects applying for contact lens fitting with a refractive error of 8.0 D sphere, with 3.0 D of astigmatism, and without clinical, topographic, or tomographic signs of keratoconus or subclinical keratoconus. After classification, the labeled images were then reviewed by a 3rd party corneal specialist who identified images with conflicting labels and adjudicated their classes by consensus. The two raters’ first-round group labels were withheld during adjudication. The dataset is comprised of 4113 images from 1371 eyes of the: normal = 500, suspect KCN = 500, and KCN = 371, and each has 3 corneal maps.

The second dataset included corneal maps from normal and KCN subjects. It was originally collected at Al-Amal eye Clinical in Baghdad Iraq, and was used in previous study as well [2]. The diagnosis of the eyes was performed by 2 experts, and patients with other ocular diseases were excluded from the study. The dataset was composed of the Pentacam Scheimpflug measurements, acquired from 213 eyes (114 normal and 99 KCN). Each eye had the 3 refractive corneal maps described in [26], which made the total images 639 images (213 × 3 maps).

### 2.2. Image Dataset Preprocessing Pipeline

**Figure 1:**
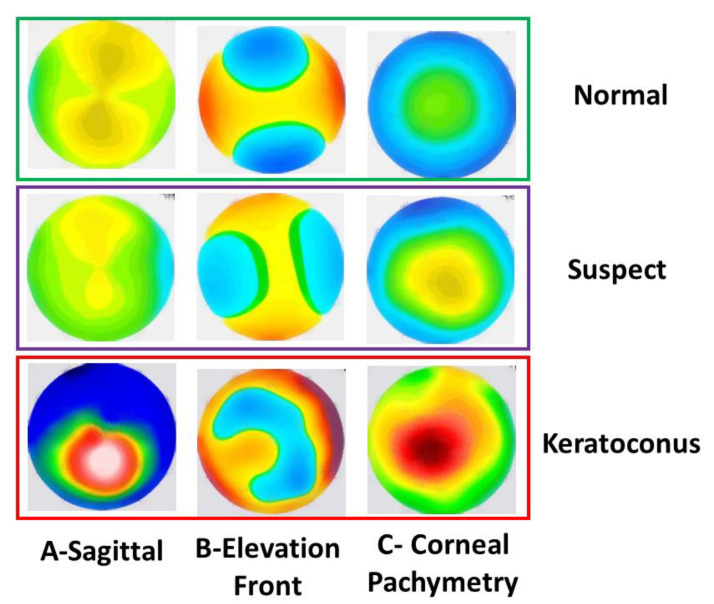
Samples of the input maps for 3 classes (normal, suspect, and keratoconus). (**A**) sagittal map; (**B**) elevation front map; and (**C**) corneal pachymetry map.

For the 3-class dataset, all maps have black numbers and annotations generated by the measurement equipment. Figure 1 shows examples of the 3 corneal maps for the 3 classes investigated in this study, i.e., normal, suspect, and KCN. All 4113 images were denoised to remove the annotations. For the independent test set, the images were clean. All images were subsequently cropped from the standard refractive maps image acquired from the Pentacam. All images from the 3-class and independent 2-class datasets (4113 + 639 = 4752) were resized to match the input of the 2 DL networks used in this study (Xception and InceptionResNetV2), i.e., 299 × 299 × 3.

### 2.3. Deep Transfer Learning

**Figure 2:**
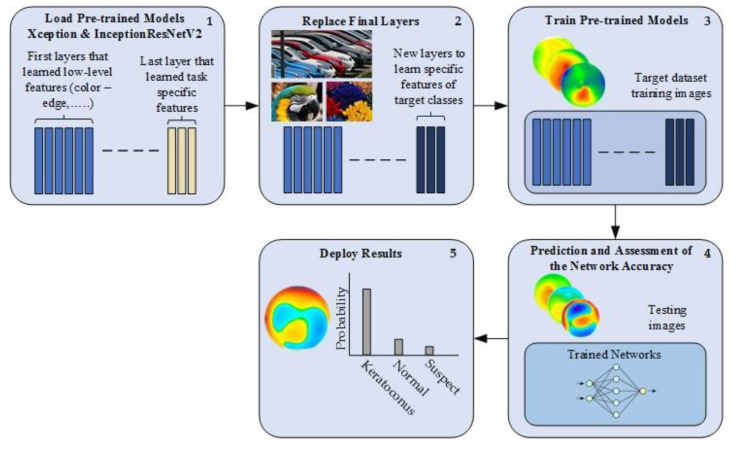
Transfer learning workflow.

Transfer learning is a technique to learn from a large dataset and then transfer the knowledge to a small dataset [27]. One of the most popular sources of transfer learning is the ImageNet [28] dataset. This dataset has 1000 classes of natural images, including various animals, flowers, objects, etc., with millions of images. Several successful applications used the ImageNet dataset as a source of transfer learning to address the issue of small datasets, including medical applications. ImageNet dataset can be useless if it does not have features relevant to the target dataset, in our case, KCN detection with corneal topography. For instance, the ImageNet dataset is a color dataset that cannot be helpful for grayscale medical images such as X-Ray, CT, MRI, etc. There is a clear mismatch between the learning features. However, our task has features relevant to the ImageNet dataset. Therefore, in this paper, two pre-trained models (Xception [24] and InceptionResNetV2 [25]) were used for this task. These two models were chosen based on their high performance with the ImageNet dataset. Both of them have an input size of 299 × 299 × 3. The workflow is described in Figure 2. To begin, the pre-trained model was loaded. Then, the last layers were replaced by new layers to learn the new features of the target dataset. The first layers learn the low-level features, such as color and edges, while the last layers typically learn the task-specific features. Therefore, the last layers are typically replaced by appropriate domain-related layers to learn the related task, which were normal, suspect, and KCN for this study, rather than 1000 classes of the initial ImageNet dataset. Next, the fine-tuned model was trained on a small dataset and tested.

### 2.4. Feature Fusion and ML Classifiers

**Figure 3:**
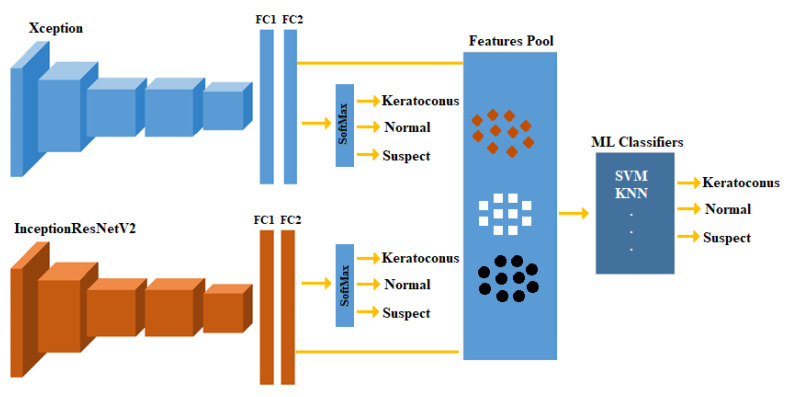
Block diagram of the proposed fusion model workflow.

Machine learning (ML) classifiers’ performance may be improved using descriptive features. Therefore, in this paper, the feature fusion technique [29] has been employed to improve the results of the individual models (Figure 3). This technique provides a compact representation (fusing) of multiple features extracted from different sources, and thus may improve the performance.

In this scenario, we trained each model (Xception and InceptionResNetV2 here) separately, then used those models to extract features from the new training and testing subsets. The extracted features from models are fused in one pool to train conventional ML classifiers. Several ML classifiers were trained and tested, including decision trees (DT), quadratic discriminant analysis, SVMs, and K-nearest neighbor (KNN). Using the classification learner application on Matlab, the best performing ML model was selected and the results were reported.

### 2.5. Training the DL Models

All used datasets were divided into three sets: training, validation, and testing. The percentage of split was equal to 80:10:10 (training, validation, and testing). The used datasets were trained in two different scenarios as follows:

- Scenario 1 (S1—2-class): training the models with two classes of normal and KCN.
- Scenario 2 (S2—3-class): training the models with three classes of normal, suspect, and KCN.

To train the networks in this study, the following settings were used. The training options included an Adam optimizer, a mini-batch size of 15, max epochs set at 100, shuffled for every epoch, and the initial learning rate was 0.001. The processor properties used in this experiment were Intel (R) Core i7/32 GB/1 TB/Nvidia RTX A3000 12 GB. Matlab 2022a was used to develop the experiments.

### 2.6. Performance Evaluation

Both the pre-trained models and the ML classifiers were evaluated based on accuracy, specificity, recall, precision, and F1-score metrics. These evaluation metrics were computed based on the TN, TP, FN, and FP values. The TN and TP implied precisely categorized negative and positive instances, whereas FN and FP denoted misclassified positive and negative cases, respectively. Every evaluation metric equation is presented as follows:

(1) $$\mathrm{Accuracy}=\frac{TP+TN}{TP+FP+FN+TN}$$

(2) $$\mathrm{Specificity}=\frac{TN}{TN+FP}$$

(3) $$\mathrm{Recall}=\frac{TP}{TP+FN}$$

(4) $$\mathrm{Precision}=\frac{TP}{TP+FP}$$

(5) $$F1_{\mathrm{score}}=2\;\times \;\frac{Precision\times Recall}{Precision+Recal}$$

In order to examine the network activations, Grad-Cams [30] were also plotted to see if the network was looking at areas of clinical importance, which is usually examined by the ophthalmologists. In addition, the learnable filters at different layers of the developed network were examined. The results were organized based on the three maps: (1) Sag, (2) ElvF, and (3) CorPac, with a test set and both scenarios, S1 (2 class, normal vs. KCN) and S2 (3 class, normal vs. suspect vs. KCN).

## 3. Results

The first dataset included 4113 corneal maps from 1371 eyes. A total of 500 eyes were normal, 500 suspect, and 371 KCN. The mean age of the participants was (normal: 36.50 ± 9.50 years, suspect: 31.80 ± 8.30 years and KCN = 31.5 ± 8.20 years, mean ± Standard Deviation, SD).

The second dataset included 639 corneal maps from 213 eyes. A total of 114 eyes were normal, and 99 KCN. The mean age of all participants was 31.4 ± 9.2 years for the normal eyes and 32.95 ± 10.86 years for KCN, mean ± SD).

### 3.1. Scenario 1: 2-Class Problem (Normal and KCN)

**Figure 4:**
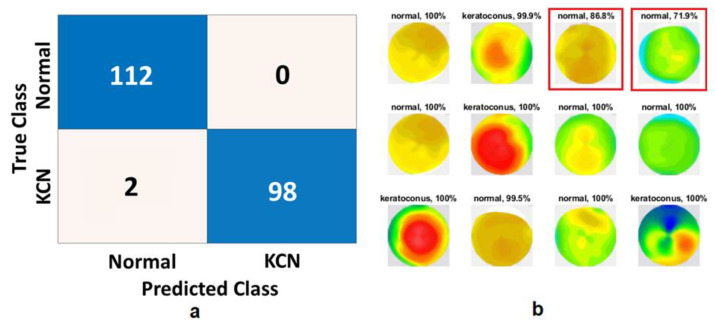
Xception model results for the Sag map with S1 (2 class). (**a**) Confusion matrix, (**b**) prediction of test set, where the red boxes are misclassified by the model.

Once we trained, tuned, and tested the model on 80/10/10 (training, validation, and testing) split of the dataset from Egypt, the Xception model generated an accuracy of 99.0% (Table 1). Only two eyes with KCN were misclassified as normal based on the Xception model (Figure 4). Once we extracted the feature using the Xception model and trained an SVM classifier, we obtained a perfect classification with 100% accuracy. As training and testing datasets were selected from the same dataset, we expected the accuracy to be high. As for the ElvF and CorPac maps, CorPac achieved the best performance with 100% accuracy with SVM.

### 3.2. Scenario 2: 3-Class Problem (Normal, Suspect, and KCN)

**Figure 5:**
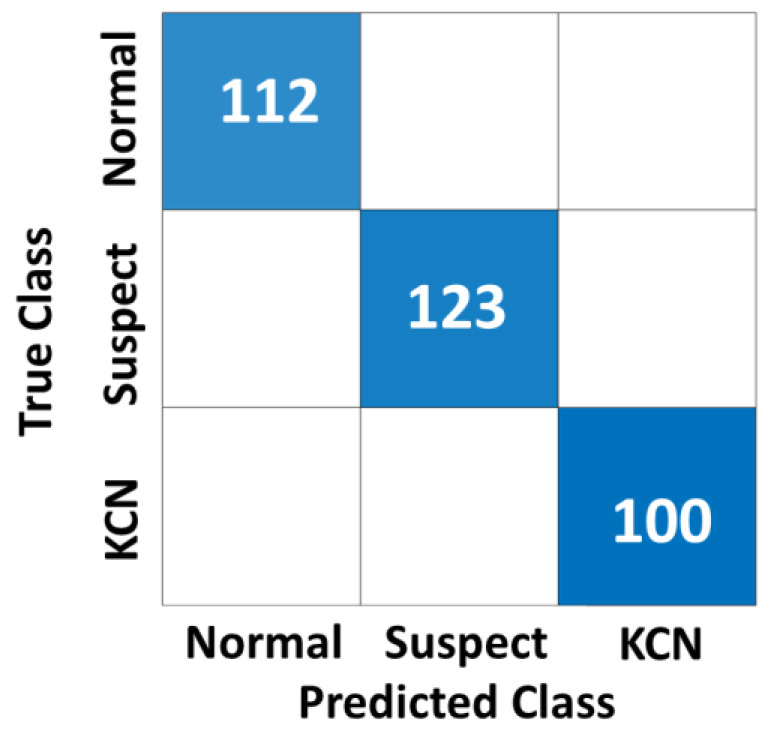
Confusion matrix for feature fusion with a decision tree for the Sag map with S2 (3 class), with an AUC of 0.99.

The DL model was trained, tuned, and tested based on the 80/10/10 split of the Egypt dataset, and the Xception model generated an accuracy of 99.7% (Table 2). The InceptionResNetV2 achieved a lower accuracy of 97.0%. Like the 2-class problem, the model with feature extraction and fusing using a decision tree obtained a perfect classification accuracy of 100%. The confusion matrix for feature fusion based on the 3-class problem (S2) is shown in Figure 5

The ElvF map will be considered in this section. Scenario 2 was first evaluated by training testing both models (Xception and InceptionResNetV2) as listed in Table 3. The Xception model achieved higher results than the InceptionResNetV2 by obtaining an accuracy of 98.5%, specificity of 98.6%, recall of 98.3%, precision of 97.5%, and F1 score of 97.9%. There are eight samples that were misclassified by the Xception model. The InceptionResNetV2 achieved an accuracy of 97.0%, specificity of 95.9%, recall of 99.1%, precision of 92.9%, and F1-score of 95.9%. There were 12 samples misclassified by the InceptionResNetV2 model.

In order to overcome the misclassified samples, features that were extracted by both models have been combined to train several ML classifiers. The highest results were achieved by two classifiers: SVM and K-nearest neighbor. SVM was chosen to be listed as shown in Table 3. SVM achieved accuracy of 99.1%, specificity of 99.0%, recall of 99.1%, precision of 98.3%, and F1 score of 98.7%.

**Figure 6:**
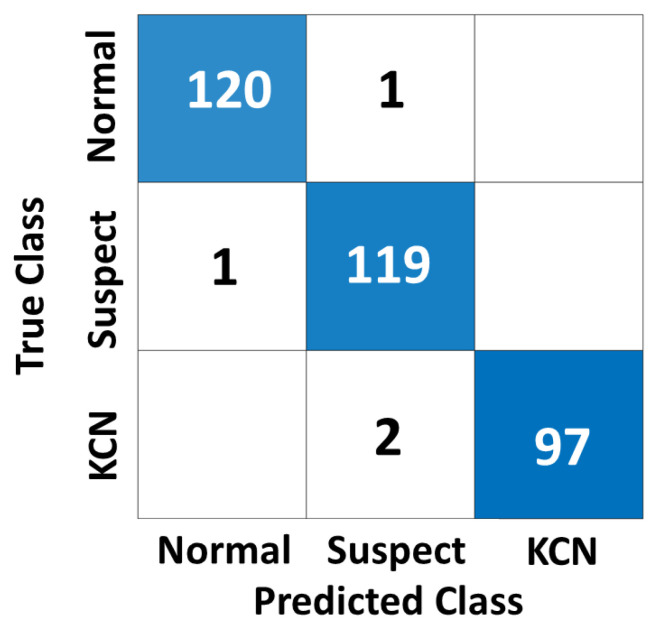
Confusion matrix of SVM for the ElvF map with the proposed DL Fusion model.

**Figure 7:**
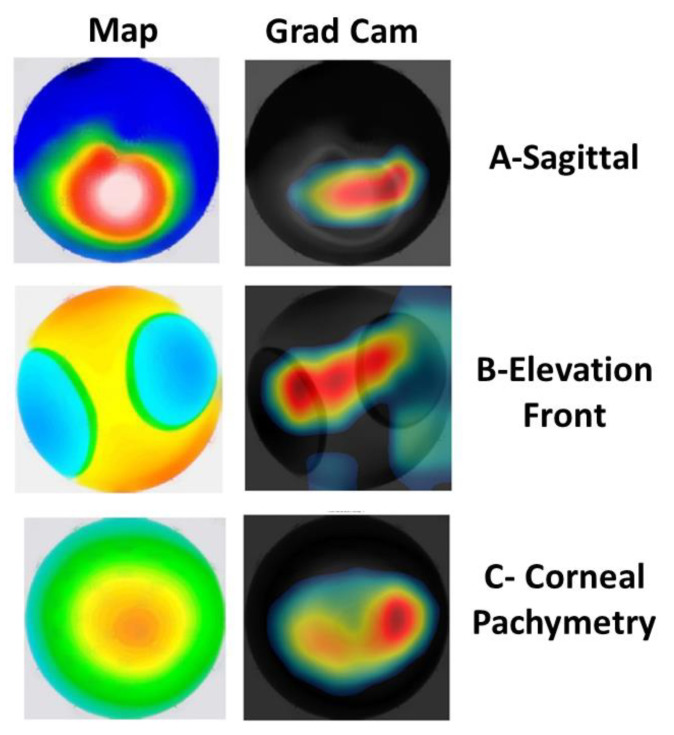
Original and class activation maps (CAMs), corresponding to different corneal maps with the Xception model for (**A**) the Sag map with KCN, (**B**) the ElvF map with a normal eye and (**C**) the CorPac map with KCN. The heatmaps show where the model focuses to make the decision.

Although the feature fusion with SVM improved the results compared to both models, four samples were misclassified, as shown in Figure 6. Lastly, the visualization of the Xception model’s focus to make decisions is shown in Figure 7B. From the obtained results, we can conclude that the model identified the appropriate place on the corneal map on which to base the decision, which validates the high achieved results.

### 3.3. Visualization of the CAMs

In order to validate the clinical relevance of the models, class activation maps (CAMs) were generated to show the regions of the corneal map that were more important for the model to make decision. Figure 7 shows the CAMs generated based on three different corneal maps including Sag, ElvF, and CorPac maps. Different CAMs suggested that the model considers clinically relevant regions of the cornea for making KCN decisions. This suggests that the model identifies the relevant spot upon which to make a decision, which, in turn, results in a correct prediction.

### 3.4. Features Extracted by the Xception Model

**Figure 8:**
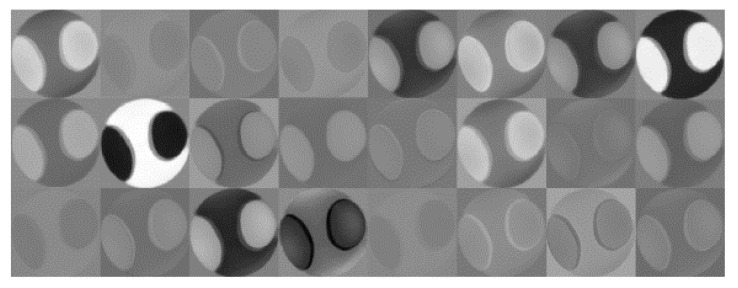
Learned filters by Xception model for the ElvF map.

To further understand how feature extraction, based on the Xception model, impacts the final KCN decision, and to assess the clinical relevance of the features, we visualized the filters that the Xception model learned from input corneal maps. Figure 8 shows the sets of filters that were learned from ElvF map. These features were essentially learned from the first convolutional layers. The shape of filters highlights the clinical relevance of the features.

### 3.5. Validating the Models Based on an Independent Dataset from Iraq

To assess how the model is generalizable to unseen corneal maps from different settings and ethnicities, we re-evaluated the performance of the model based on an independent dataset. This dataset was collected from an eye clinic in Baghdad, Iraq, which represents corneal data from different settings with likely different settings. Table 4 shows the accuracy metrics of the 2-class problem based on three different corneal maps.

**Figure 9:**
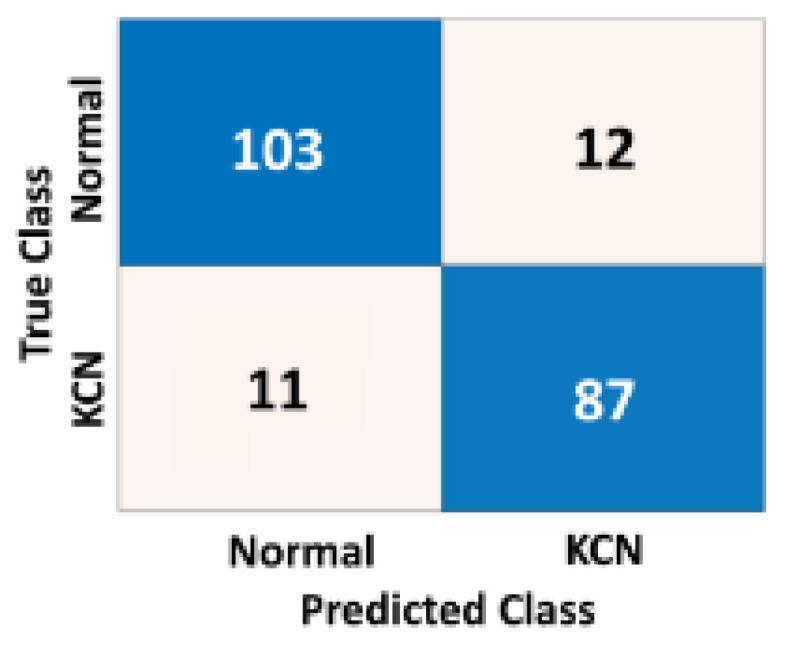
Confusion matrix of the independent test set for the *Sag* map, where the AUC is equal to 0.91.

**Figure 10:**
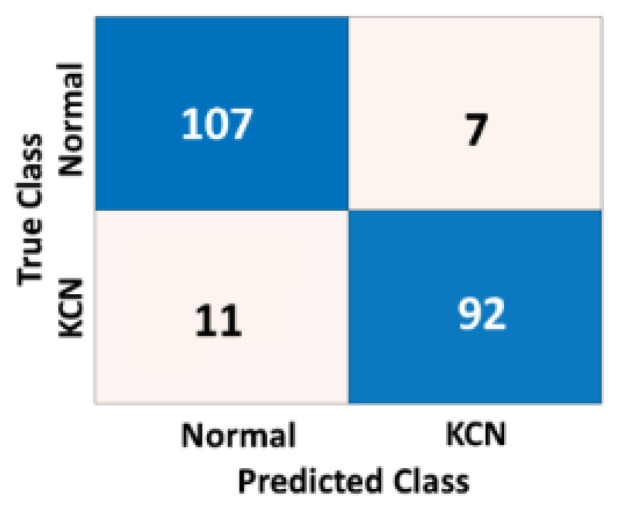
Confusion matrix of the independent test set for the *ElvF* map, where the AUC is equal to 0.96.

**Figure 11:**
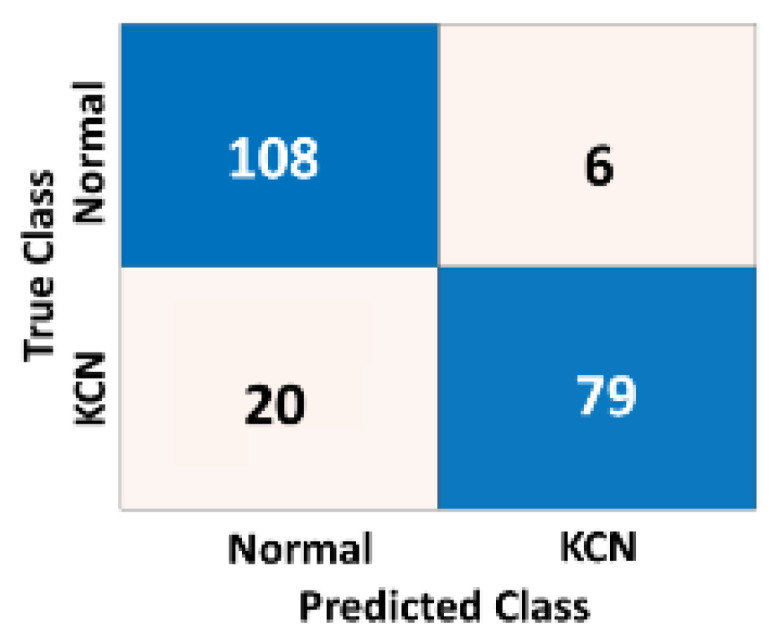
Confusion matrix of the independent test set for the *CorPac* map, where the AUC is equal to 0.92.

Figure 9, Figure 10 and Figure 11 show the confusion matrices of the fused CNN model to discriminate normal from KCN based on three different maps: Sag, ElvF, and CorPac, respectively. The model based on corneal ElvF map generated the highest AUC of 0.96, with an accuracy of 91.7%.

## 4. Discussion

We developed several conventional CNN models, based on state-of-the-art architectures, including Xception and InceptionResNetV2, which were able to detect corneas with suspect or established KCN. We observed that the accuracy of these CNN architectures to identify normal eyes from eyes with established KCN based on different corneal maps, reached around 99%. A similar efficacy was observed in the same CNN models for distinguishing normal eyes, eyes with suspect KCN, and eyes with established KCN. While these results were highly promising, the testing subset was selected from the same dataset as the model training.

To assess the generalizability, we evaluated the accuracy of the CNN models based on another independent dataset collected from a Pentacam instrument in another country, with likely different device settings. For the S1 (two-class) problem, we achieved an AUC and accuracy of 0.99 and 100, respectively, with the CorPac map. For the S2 (three-class) problem, we obtained an accuracy of 100 with the Sag map. Our findings suggest that our CNN models are generalizable to unseen corneal maps from other instruments from other countries with likely different settings, and corneal maps from different populations.

With our DL fusion models (Xception and InceptionResNetV2), different classifiers were utilized including DT, quadratic discriminant analysis, KNN, and SVM. SVM and DT were the best performing classifiers compared to the other ML models, with an accuracy of 99.1 and 100, respectively.

Examining Table 1, it can be noted that the CorPac map generated the highest performance. For testing with the independent test dataset, the ElvF map was the best map, with an AUC of 0.96 and accuracy of 91.7% (Table 4).

Based on the results in Table 3 and Table 4, the InceptionResNetV2 model h lower performance than that of Xception model. In the case of InceptionResNetV2, the low number of parameters could be due to a few reasons. One possibility is that the model was fine-tuned or pruned to reduce its size and complexity. Another possibility is that the dataset used to train the model was relatively small or simple, and therefore, a smaller model was sufficient to achieve good performance as in the case of Xception model.

A comparison of our model with other models previously published is shown in Table 5. It should be noted that suspect KCN was not included in many previous studies [2,13,15,19,22]. To tackle that, we developed and validated our proposed method on the three-class problem, including suspect KCN. For example, the model previously introduced by Al-Timemy et al. [23] includes an ensemble of deep transfer learning to combine the four topographic maps with Pentacam indices, whereas our proposed fusion model dealt with each topographic map individually, and achieved good performance for each map.

As shown in the learnable filters displayed in Figure 8, the (Xception) model learned very strong features from the ElvF map. Additionally, the results from the GradCam, shown in Figure 7B, illustrate that the model is focusing on an area at the center, which is the clinically relevant region. The accuracy obtained with the Xception model was equal to 98.5%. This may explain the good performance achieved with the Xception model.

While we used relatively large datasets of corneal maps from two datasets collected from two different countries, additional datasets with larger number of corneal maps from other ethnicities/races are desirable to further validate the proposed CNN models. Additionally, our CNN models are limited to corneal data from Pentacam. Therefore, the use of innovative device-agnostic CNN models is warranted to exploit data from other corneal topography instruments, such as CASIA, Orbscan, and Galilei.

## 5. Conclusions

DL fusion-based methods were proposed and developed for KCN detection. While single CNN architecture achieves good performance, the fusion of two DL models (Xception and InceptionResNetV2) with features from these models and an SVM classifier is particularly suited to meet the challenge of identification of earlier stage, clinical suspect KCN, distinct from established KCN. We validated our proposed method on a three-class dataset and achieved an accuracy of 97–100%. Furthermore, independent evaluation of a two-class dataset from a different country showed an accuracy of 88–92% and an AUC 0.91–0.96 for the three corneal maps.

## Figures and Tables

**Table 1:** Performance metrics of the Xception model applied on Sag, ElvF, and CorPac maps based on scenario S1 (2 class), without and with SVM.

Corneal Map	DL Model	Accuracy	Specificity	Sensitivity	Precision	F1-Score
Sag	Xception	99.0	98.0	100	98.2	99.12
	Xception + SVM	100	100	100	100	100
ElvF	Xception	95.9	92.0	99.1	93.7	96.3
	Xception + SVM	95.9	92.1	99.1	93.7	96.4
CorPac	Xception	100	100	100	100	100
	Xception + SVM	100	100	100	100	100

**Table 2:** Results of the Sag map on the S2 (3 class) scenario, with test set with a decision tree classifier.

DL Model	Accuracy	Specificity	Recall	Precision	F1-Score
Xception	99.7	99.5	100	99.1	99.6
InceptionResNetV2	97.0	95.3	100	92.4	96.0
Feature Fusion + Decision Tree	100	100	100	100	100

**Table 3:** Results of the ElvF map on S2 (3 class) scenario with the test set.

DL Model	Accuracy	Specificity	Recall	Precision	F1-Score
Xception	98.5	98.6	98.3	97.5	97.9
InceptionResNetV2	97.0	95.9	99.1	92.9	95.9
Feature Fusion+ SVM	99.1	99.0	99.1	98.3	98.7

**Table 4:** The results of all maps for the independent test set from Iraq, with a feature fusion of Xception and InceptionResNetV2.

Corneal Map	Accuracy	Specificity	Sensitivity	Precision	F1-Score	AUC
Sagittal	89.2	90.3	87.8	88.7	88.3	0.91
Elevation Front	91.7	90.6	92.9	89.3	91.0	0.96
Pachymetry	87.7	84.3	92.9	79.8	85.8	0.92

**Table 5:** The previous literature on KCN detection with deep learning.

Study	Imaging Device	Number of Classes	Number of Maps	Network Used	Comments	Accuracy
Zéboulon et al. [15]	Orbscan	2	3000	CNN	No suspect	98.3%
Al-Timemy et al. [2]	Pentcam	2	534	Ensemble of Alexnet	No suspect	95–98.3%
Chen et al. [19]	Pentcam	2	1926	VGG16	No suspect	97.85%
Lavric and Valentin [20]	SyntEyes and SyntEyes KTC models/1 map		3000	KeratoDetect	No suspect synthetic maps	99.3%
Kuo et al. [13]	Tomy TMS-4 Topographer	2	354	VGG16 InceptionV3 ResNet152	No suspect	93.1% 93.1% 95.8%
Firat et al. [22]	Pentcam	2	628	ALexNet and SVM	No suspect	98.53%
This study	Pentcam	3	4113	Fusion of Xception and InceptionResNetV2	-	97–100%

## Data Availability

The image dataset and the trained models and Matlab codes are made available from the link: https://drive.google.com/drive/folders/1RiiAsDTmpNTqcCktuZPRUmh2iElB_ACs?usp=sharing (accessed on 28 February 2023).
